# Managing tricuspid valve pathology in multiple valvular heart disease

**DOI:** 10.1016/j.amsu.2022.104719

**Published:** 2022-09-15

**Authors:** Denny Suwanto, Ivana Purnama Dewi, Mohammad Budiarto

**Affiliations:** a Faculty of Medicine, Airlangga University, Surabaya, Indonesia; b Cardiology and Vascular Medicine Department, Dr. Soetomo General Hospital, Surabaya, Indonesia; c Faculty of Medicine, Duta Wacana Christian University, Yogyakarta, Indonesia

**Keywords:** Multivalvular heart disease, Tricuspid valve, Diagnosis, Therapy

## Abstract

**Introduction:**

Multiple valvular heart disease (MVD) is a general term to describe regurgitant and stenotic combination involving the same valve and/or occurring in ≥2 cardiac valves. Limited data and paucity in guidelines render the diagnosis and management. This article aims to provide a state-of-the-art review concerning the diagnosis and management strategies of MVD.

**Case presentation:**

We report a 46-year-old female with worsening dyspnea and fatigue. We perform multiple echocardiography parameters. We diagnose patients with the stenotic mitral valve, stenotic-regurgitant aortic valve, and stenotic-regurgitant tricuspid valve (TV). Double mechanical valve replacement and TV commissurotomy with Kay procedure were done with excellent results.

**Clinical discussion:**

The prevalence of MVD is 15% in those undergoing cardiac surgery; however only 1% of those who underwent triple valve surgery involve TV. The presence of TV lesion may complicate the natural history, clinical presentation, management, and outcomes. Echocardiography with valid method remains an important tool in assessment of patients with MVD. Multidiscipline heart team discussion is essential in determining individual risk, appropriate management methods, and long-term survival.

**Conclusion:**

The expertise of multidisciplinary heart valve team is of utmost importance in determining diagnosis and optimal management strategy.

## Highlights

• Multivalvular heart disease (MVD) is a complicated condition. A regurgitant and stenotic combination involves the same valve and/or occurs in ≥2 cardiac valves in MVD cases.
• The presence of tricuspid valve (TV) lesion may complicate the natural history, clinical presentation, management, and outcomes.
• Echocardiography is an important modality that can help to diagnose and sometimes can provide a prognosis after surgery.

## 1 Introduction

Multivalvular heart disease (MVD) is concurrent stenosis and regurgitation involving more than 2 cardiac valves [1]. Euro Heart Survey reported that MVD involved 15% of those who underwent valvular surgery [2]. Data from the Society of Thoracic Surgeons from 600.000 individuals underwent valvular heart surgery, 11% underwent multivalvular surgery. From that, 58% mitral-aortic surgery, 31% mitral-tricuspid surgery, 3% aorta-tricuspid surgery, and 8% triple valve surgery [3]. Tricuspid valve (TV) pathology is common finding either as a primary or secondary pattern. Despite its severity and pattern, tricuspid pathology significantly increased mortality by 25% [4]. Therefore, understanding the management strategy of tricuspid pathology is an essential part of MVD management.

## 2 Case report

We present a single case report and has been reported in line with SCARE 2020 criteria [5]. A 46 years-old female came to emergency unit with a chief complaint of dyspnea and fatigue on daily activities. Similar symptoms have been experienced since her late 30's and worsening over the past two years. There was no history of previous heart disease, family history, or genetic information. Physical examination revealed distended neck vein, irregular heart rate, with grade III/IV diastolic murmur at the apex with clear lung. The liver was mildly palpable, with no lower extremities pitting edema. There was atrial fibrillation on electrocardiography and cardiomegaly on chest X-Ray.

**Fig. 1:**
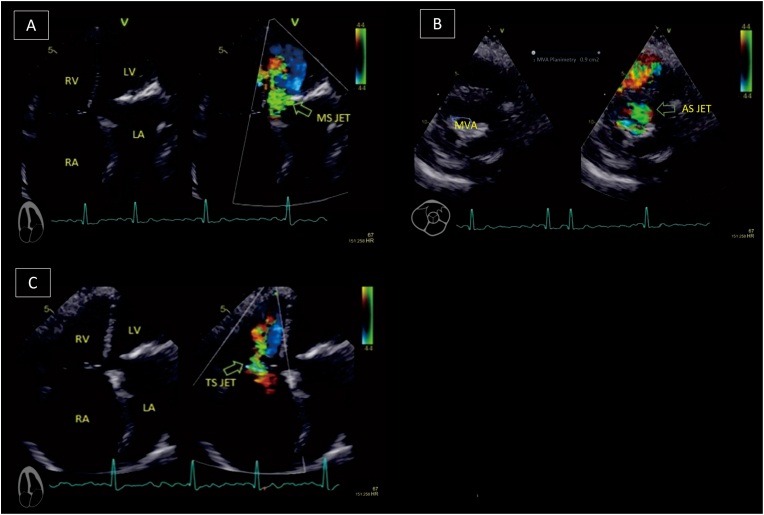
Transthoracal echocardiography showed (A) mitral stenosis in 4 chamber view, (B) The mitral valve area planimetry was 0.9cm^2^ from parasternal short axis view, (F) from 4 chamber view it's seen tricuspid stenosis.

**Fig. 2:**
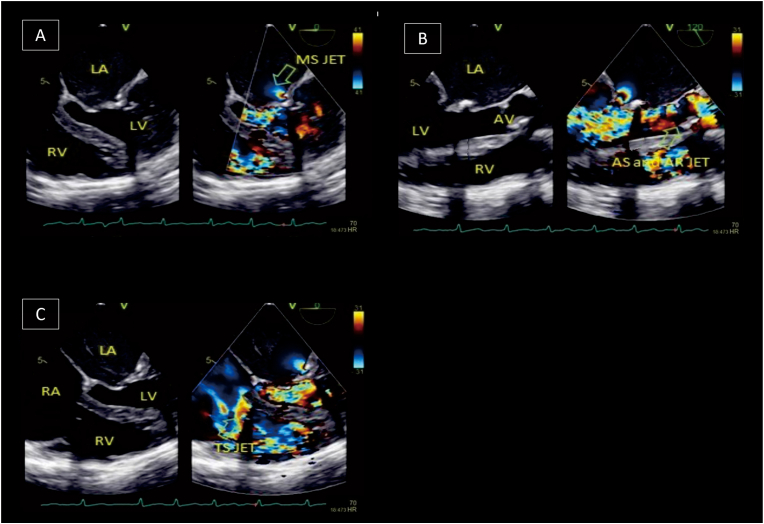
Transesophageal echocardiography showed (A) mitral stenosis jet, (B) tricuspid stenosis jet, and (C) aortic stenosis with aortic regurgitation jet.

Echocardiography evaluation performed by an echocardiography expert revealed (Fig. 1 and Fig. 2); severe mitral stenosis (MS) (Mitral Valve Area planimetry 0.9cm^2^, Wilkins score 2-3-2-3), a calcified aortic valve (AV) with severe aortic stenosis (AS) (AV VMax 4.62 m/s; Aortic Valve Area planimetry 0.6cm2; AV mean Pressure Gradient (PG) 51.91 mmHg; Stroke Volume Index 47.17ml/m2), moderate aortic regurgitation (AR) (Vena Contracta Width (VCW) 0.4cm, Jet width/Left Ventricular Outflow Tract (LVOT) width 26%, Jet Cross-Sectional Area (CSA)/LVOT CSA 17.6%), severe tricuspid stenosis (TS) (TVmeanPG 9.33 mmHg, Inflow Velocity Integral 65.4cm), moderate tricuspid regurgitation (TR) (TRmaxPG 61.52 mmHg), and dilatation of all chambers, LV Ejection Fraction 61%, and concentric remodeling of the LV.

**Fig. 3:**
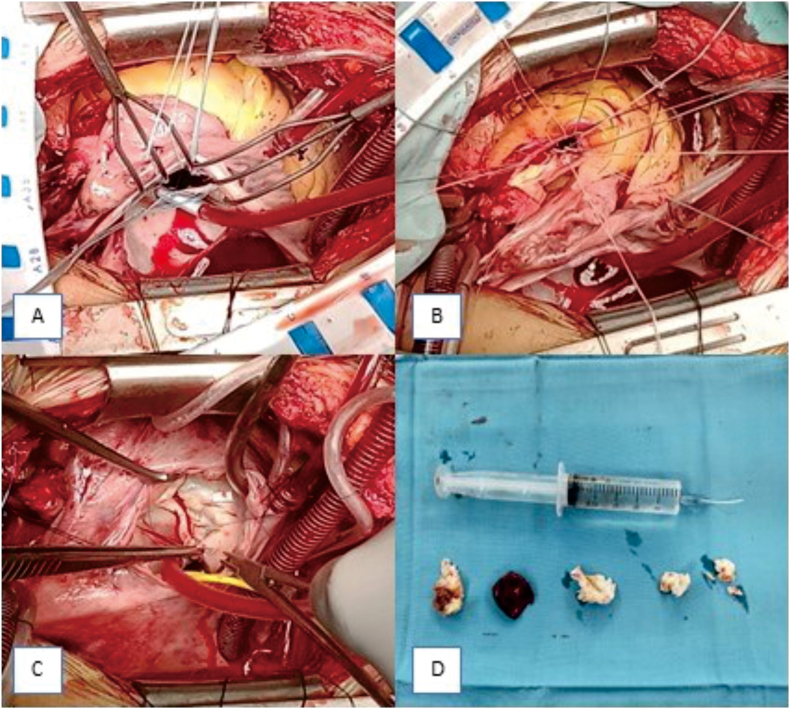
(A) Mitral valve surgery; (B) Aortic valve surgery; (C) Tricuspid valve surgery; (D) Post-surgery valvular and thrombus evacuated from left atrium.

We diagnose patients with stenotic mitral valve with severe calcification, stenotic-regurgitant aortic valve, and stenotic-regurgitant tricuspid valve with moderate pulmonary hypertension (PH). Double mechanical valve replacement (DVR) at the aortic and mitral valve (St Jude mechanical valve, Saint Paul, Minn, leaflet pyrolytic carbon valve), and TV commissurotomy with Kay procedure were done by a senior thoracic and cardiovascular surgeon with excellent results. A 4cm × 1.5cm x 2.2cm thrombus from left atrium was evacuated (Fig. 3).

Postoperative echocardiography evaluation showed: mechanical mitral valve (MV) (St.Jude 29mm) with good position and function (Peak Velocity 1.30 m/s, Mean Gradient 3.33 mmHg, Velocity-Time Integral (VTI) prosthetic mitral valve/VTI LVOT 1.46, Effective Orifice Area (EOA) 2.5cm2, MV mean Pressure Half Time (PHT) 68 ms) and mechanical AV (St.Jude 27mm) with good position and function (Peak Velocity 1.62 m/s, Mean Gradient 4.93 mmHg, Doppler Velocity Index 0.91, EOA 3.2cm2, Acceleration Time 72 ms), moderate TS (TV Area 1.11cm2; TV VTI 33.6cm; TVmeanPG 3.47 mmHg; TV PHT 171 ms), and trivial TR (TRmaxPG 20.20 mmHg).

The patient was given furosemide 20 mg once daily, bisoprolol 2,5 mg once daily, and heparin with aPTT targeted to 2–2.5 times control. After four days of stabilization on the intensive care unit, the patient feels relieved after the operation because the complaints are much reduced. She was then transferred to standard care and discharged two days later with long-term anticoagulant switched to warfarin with an INR target of 2.5–3. The patient was followed up every month for 1 year. The patient conditions in getting better, she can carry out normal activities without any complaints.

## 3 Discussion

Multivalvular heart disease induces a complex hemodynamic interaction that worsens and masks the clinical presentation of coexisting valvular lesions. The combination of MS and AS will reduce the end-diastolic volume, flow rate, and pressure gradient [6]. LV hypertrophy, diastolic dysfunction from AS, and increased left atrial pressure from MS will induce post-capillary PH and secondary TR. The presence of TR significantly reduces survival by 64–90% [7]. Long-term TR will induce dilatation of tricuspid annulus and decreased right ventricular function, both known as a predictor of worse postoperative outcomes [8,9].

The presence of TR significantly reduces survival by 64–90% vary on its severity [8]. Functional tricuspid regurgitation complicates MVD in 90% cases. It arises from tricuspid annular dilatation induced by RV remodeling secondary to passive pulmonary hypertension [9.10]. It is important to assess the RV function to measure the consequences of tricuspid regurgitation, as reduced RV function is an indicator of poor prognosis in those undergoing valvular heart surgery. Several RV function parameters are commonly measured, such as RV Index of Myocardial Performance (RIMP) or Tei Index (cut-off abnormal >0.43 with pulsed Doppler dan >0.54 measured with tissue Doppler), Tricuspid Annular Plane Systolic Excursion (TAPSE) (cut-off abnormal <17 mm), Fractional Area Change (FAC) (cut-off abnormal <35%) and tissue Doppler-derived tricuspid lateral annular systolic velocity (cut-off abnormal <9.5 cm/s).

As in our case, TS in the context of mitral and aortic stenosis suggests rheumatic etiology. The mitral and aortic stenosis pattern in our case fulfills the WHO criteria of rheumatic valve disease. Therefore, we hypothesize the same etiology also caused TS present in our case. Echocardiography parameter showed severe TS (TV mean Pressure Gradient 9.33 mmHg, plethoric inferior vena cava). However, TR was most likely secondary to her left-sided valve disease-inducing post-capillary pulmonary hypertension, proven by dilatation of TV annular diameter by 48 mm. Our patient's clinical manifestation was a mixture of left-sided valves disease (dyspnea and fatigability) and tricuspid stenosis-regurgitation (abdominal discomfort, palpable liver, distended neck vein).

Determining the severity of valvular pathology is an important part of the diagnosis. Hemodynamic consequences of MVD will affect blood flow, size, and LV function, affecting the quantification of valvular severity (1). Several echocardiographic quantifications such as continuity equation and PHT are invalid in the presence of MVD and abnormal LV [8]. The planimetry area is the recommended quantification method for the mitral and aortic valve in the MVD case [10]. Calculation of AR severity using PHT and deceleration slope method was not reliable in the presence of MS and prolonged LV filling time. Therefore AR, in this case, is best assessed using a semiquantitative parameter such as VCW, jet width/LVOT width, and jet CSA/LVOT CSA [11,12].

There are limited recommendations and studies on MVD. Most recommendations by ACC/AHA, ESC, and EACTS are classified with the level of evidence C. Correction of MS without correction toward AS or planned as a staged procedure will deliberate dismal outcome. Non-compliant and thickened LV when receiving a sudden increase of LV end-diastolic volume from corrected MS and poor afterload from uncorrected AS may cause an acute increase of LVEDP, when transmitted backward to lung, will induce lung congestion edema [[13], [14], [15]].

Echocardiography in our patient showed a non-thickened TV leaflet with preserved subvalvular apparatus indicating non-severe calcification. Therefore commissurotomy was chosen over valvular replacement [[13], [14], [15]]. Secondary moderate TR with a dilated annulus concurrently with left-sided valve disease was also indicated for surgery. Annuloplasty with Kay procedure was performed to correct TR [9,16]. In this case, severe calcification (Wilkins score 10) with co-incidence of severe AS, moderate AR, TS, and TR, rendering percutaneous mitral commissurotomy. Therefore, DVR of the mitral and aortic valve was chosen with long-term anticoagulation using warfarin.

## 4 Conclusion

Multiple valvular heart disease is a prevalent valvular pathology, with RHD, particularly in a developing country. A combination of valvular pathology will produce complex hemodynamic interaction with a particular challenge in diagnosis, determining pathology severity, and management. Multidiscipline heart team discussion is essential in determining individual risk, appropriate management method, and long-term survival.

## Provenance and peer review

Not commissioned, externally peer-reviewed.

## Ethical approval

The authors received no financial support for the research, authorship, and/or publication of this article.

## Sources of funding

The authors received no financial support for the research, authorship, and/or publication of this article.

## Author contributions

Denny Suwanto: attending physician, data collection, writing the paper.

Ivana Purnama Dewi: writing the paper, editing the manuscript for publication.

Mohammad Budiarto: supervision.

## Registration of research studies

N/A.

## Guarantor

Denny Suwanto.

## Consent

Written informed consent was obtained from a legally authorized representative for anonymized patient information to be published in this article.

## Declaration of competing interest

The authors declare that there is no conflict of interest.
